# Yellow Fever Not Personally Communicable

**Published:** 1879-08

**Authors:** 


### YELLOW FEVER NOT PERSONALLY COMMUNICABLE.

No question involves more serious results than that touching the manner in which yellow fever is contracted. We have repeatedly urged the importance of correctness in public opinion on this point, and now insist that medical men lay aside pre-conceived, long-cherished views, and take facts—stubborn facts—upon which to form opinion for public expression. Upon the opinion of prominent physicians, speaking and acting by authority of municipal, State and United States governments, the people are inclined to rely, and hence that opinion should be based on something more than mere theory, however long ago promulgated.

On previous occasions, and repeatedly, we have expressed the opinion that error in regard to the origin of fever is a fruitful source of its wide-spread devastation.

The almost universal opinion expressed on this subject, by talking and writing people, is, that yellow fever comes to this country by importations of "germs," as fomites in clothing and merchandise, or in the person of a subject of the disease, and is transported along the thoroughfares of commerce and travel in the same way throughout the country. Confidence in this doctrine promulgated by government medical officials and backed by half a million of dollars, induces the citizens of places where yellow fever occurs, to expect protection against it by quarantine. Thus the people of Memphis, under this false security, waited amid the poisonous effluvia from the marshes on the west and stagnant bayou on the east, until the malaria had done its work of death. Sad and painful is the thought that the large sum of money appropriated by Congress has thus had the effect of destroying many helpless citizens of that once flourishing city.

In the light of the recent convincing experience of

Memphis, will those entrusted by the Government with the duty of protecting the people against the fever, persist in acting on a theory so signally disproved?

While importation and transportation were officially announced as giving origin to the fever in 1878—but without satisfactory proof—the present prevalence is so undoubtedly of local origin, that the national board and quarintinists generally are compelled to admit it. Why then spend the half million of dollars on quarantine? It is now proven of no value, and therefore, to say nothing of the loss of life from false security it may give those silly enough to rely on it hereafter, we say, in the language of the Memphis *Appeal*, "the quarantinists may step down and out."

---

The Southern Advertising Agency that is being organized by Mr. Francis Fontaine, promises to be a very commendable and beneficial enterprise. Journals and periodicals throughout the South will find it to their interests to cooperate and patronize this agency—full particulars of which will be furnished by Francis Fontaine, No. 34, Park Row, New York.

Already has the agency received much encouragement by the cooperation of several Southern papers. By instituting Mr. Fontaine sole advertising agent, the individual paper reserves the right to accept advertisements from advertisers direct.

We hope the enterprise will be encouraged to the fullest.

